# Recent advances in the plant epitranscriptome

**DOI:** 10.1186/s13059-023-02872-6

**Published:** 2023-03-07

**Authors:** Lisha Shen, Jinqi Ma, Ping Li, Yujin Wu, Hao Yu

**Affiliations:** 1 Temasek Life Sciences Laboratory, 1 Research Link, National University of Singapore, Singapore, 117604 Singapore; 2 Department of Biological Sciences, Faculty of Science, National University of Singapore, Singapore, 117543 Singapore

## Abstract

Chemical modifications of RNAs, known as the epitranscriptome, are emerging as widespread regulatory mechanisms underlying gene regulation. The field of epitranscriptomics advances recently due to improved transcriptome-wide sequencing strategies for mapping RNA modifications and intensive characterization of writers, erasers, and readers that deposit, remove, and recognize RNA modifications, respectively. Herein, we review recent advances in characterizing plant epitranscriptome and its regulatory mechanisms in post-transcriptional gene regulation and diverse physiological processes, with main emphasis on *N*^6^-methyladenosine (m^6^A) and 5-methylcytosine (m^5^C). We also discuss the potential and challenges for utilization of epitranscriptome editing in crop improvement.

**Supplementary Information:**

The online version contains supplementary material available at 10.1186/s13059-023-02872-6.

## Introduction

A variety of naturally occurring chemical modifications on cellular RNAs, collectively termed as epitranscriptome, add an additional layer of regulatory information to RNAs. To date, there are over 170 distinct RNA modifications discovered, with information stored in the MODOMICS database [1]. Over the last decade, advances in techniques in detecting RNA modifications and technologies coupling the next generation sequencing with antibody, chemical, and enzymatic approaches in mapping RNA modification sites have profoundly improved our understanding of the complexity and function of epitranscriptome, especially in messenger RNAs (mRNAs). Thus far, diverse mRNA modifications have been discovered and mapped in eukaryotic cells, including *N*^7^-methylguanosine (m^7^G) and nicotinamide adenine diphosphate (NAD^+^) modifications [2] at the 5′-cap, and other modifications occurring internally, such as *N*^6^-methyladenosine (m^6^A) [3, 4], *N*^1^-methyladenosine (m^1^A) [5, 6], 5-methylcytosine (m^5^C) [7], *N*^4^-acetylcytidine (Ac4C) [8], 5-hydroxymethylcytosine (hm^5^C) [9], m^7^G [10], and pseudouridine (ψ) [11, 12]. Accumulating evidence suggests that these modifications with great chemical and structure diversities provide extraordinary regulatory potential in modulating RNA metabolism, thus affecting gene expression. Moreover, extensive characterization of their effector proteins, including writers, erasers, and readers that perform respective functions in installing, removing, and decoding mRNA modifications, has also significantly advanced our knowledge in epitranscriptome regarding its fundamental regulatory roles in diverse and dynamic cellular processes.

**Fig. 1:**
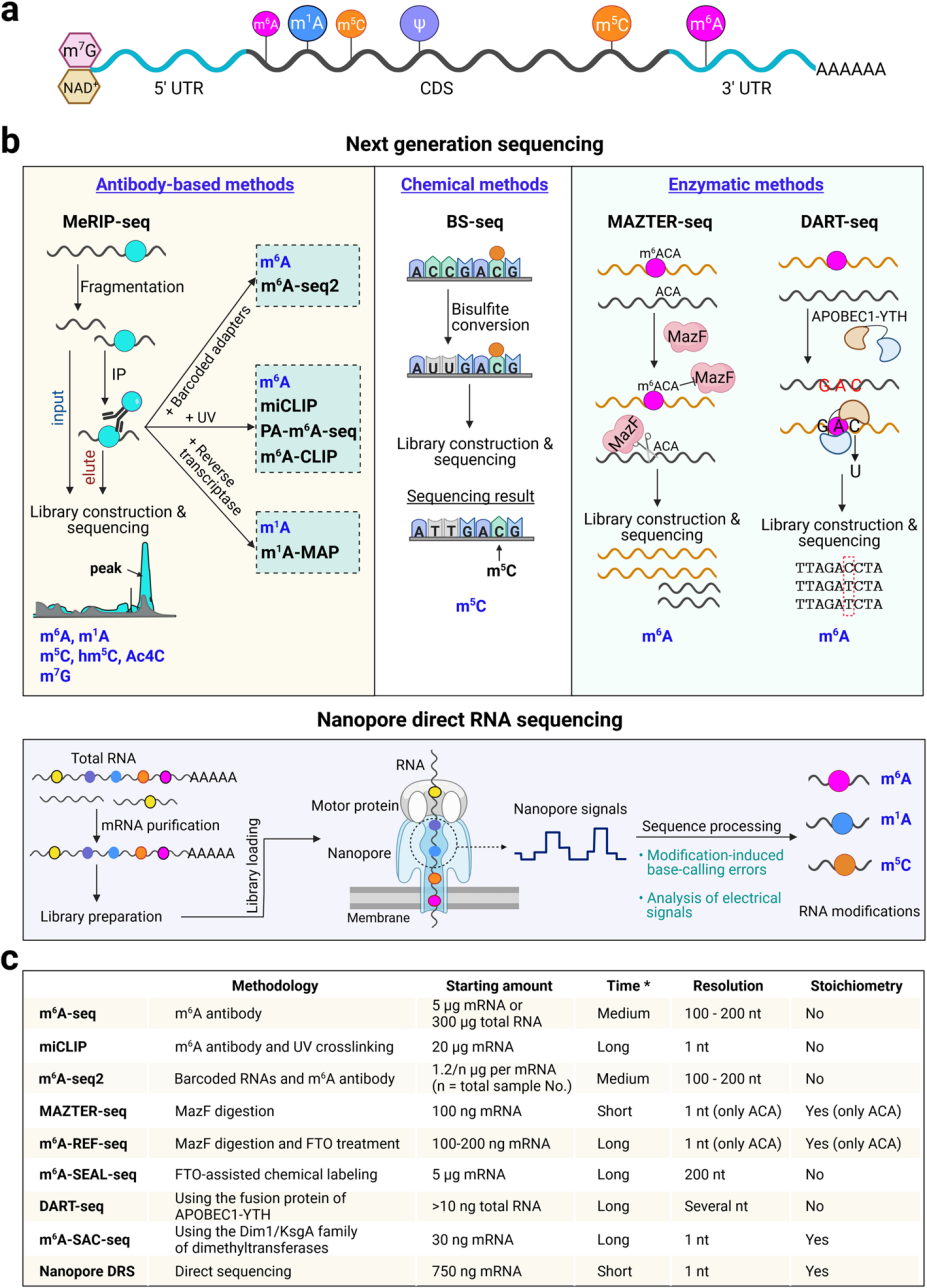
An overview of the plant epitranscriptome and techniques for mapping epitranscriptome. **a** Known RNA modification in plants. Predominant locations of various RNA modifications are illustrated in a transcript. The mRNAs are capped with *N*^7^-methylguanosine (m^7^G) or nicotinamide adenine diphosphate (NAD^+^) and contain internal modifications, including *N*^6^-methyladenosine (m^6^A), *N*^1^-methyladenosine (m^1^A), 5-methylcytosine (m^5^C), and pseudouridine (ψ), in plants. **b** Approaches for mapping RNA modifications. Techniques coupling next generation sequencing with antibody-, chemical-, and enzyme-based approaches are shown in the upper panels, while nanopore direct RNA sequencing is illustrated in the lower panel. **c** Comparison of the key features of different m^6^A profiling techniques. The asterisk indicates the time required for sample preparation before library construction and sequencing. Created with Biorender.com

In plant epitranscriptome, m^6^A represents the most prevalent and best characterized internal modification on mRNA. Its landscape and the relevant effectors have been revealed across a variety of plant species [13–16]. Several other internal modifications, including m^5^C, m^1^A, and ψ, have been mapped on a transcriptome-wide scale in some plant species along with identification of their writers (Fig. 1a) [17–19]. In addition to these internal modifications, plant mRNAs are also modified at their 5′ end, such as the canonical m^7^G or non-canonical NAD^+^ caps (Fig. 1a) [20, 21]. In this review, we highlight recent advances in our understanding of plant epitranscriptome and its regulatory mechanisms in post-transcriptional gene regulation and physiological processes, with main emphasis on m^6^A and m^5^C. We also highlight the outstanding questions pertaining to plant epitranscriptome and discuss the potential and challenges of future crop improvement through epitranscriptome editing.

## Advances in epitranscriptome profiling technologies

The breakthrough of epitranscriptome studies in the past decade is largely attributed to advancement in epitranscriptome detecting and profiling technologies. Biochemical methods based on physicochemical properties like liquid chromatography-tandem mass spectrometry (LC-MS/MS) [22, 23] allow precise detection and quantification of overall levels of multiple epitranscriptomic marks in plants, including m^6^A [24], m^1^A [17], m^5^C, and hm^5^C [19]. Dot blot analysis using antibodies recognizing specifically modified nucleotides also detects overall RNA modification levels with low sensitivity and precision [25]. However, these approaches are unable to profile the context-dependent transcriptome-wide pattern of RNA modifications. To fill this gap, a variety of high-throughput transcriptome-wide profiling technologies have been developed, including those coupling short-read based sequencing with antibody, chemical, and enzymatic methodologies and Nanopore long-read direct RNA sequencing.

### Antibody-, chemical-, and enzyme-based epitranscriptome profiling

Methods coupling next generation sequencing with antibody immunoprecipitation of modified RNAs, including m^6^A-seq [3, 4], m^1^A-seq [6], m^5^C-seq [26], hm^5^C-seq [9], Ac4C-seq [8], and m^7^G-seq [10] (Fig. 1b), are most commonly used so far for mapping various RNA modifications. These methods have significantly contributed to our current knowledge of the location and distribution of epitranscriptome marks. For instance, m^6^A-seq has revealed the prevalence of m^6^A methylation in thousands of transcripts with unique and conserved distribution preferentially around stop codons and in 3′ untranslated regions (UTRs) in eukaryotic organisms [3, 4, 27–29]. Nevertheless, these approaches suffer from poor resolution (100–200 nt) on positional information of epitranscriptome marks. Additional steps have been introduced to improve these methods for detecting RNA modifications at single-base resolution. First, incorporating a UV crosslinking step to RNA immunoprecipitation as shown in m^6^A individual-nucleotide-resolution crosslinking and immunoprecipitation (miCLIP) [30, 31], m^6^A crosslinking immunoprecipitation (m^6^A-CLIP) [32], and photo-crosslinking-assisted m^6^A sequencing strategy (PA-m^6^A-seq) [33] improves m^6^A detection to the exact or + 1 site. However, these methods are unable to quantify differential modifications among samples without utilizing methylated spike-in controls or an input library for correction or normalization [34]. Second, specific reverse transcriptases are applied to improve the detection resolution for RNA modifications that induce misincorporations during reverse transcription. These methods include m^1^A-MAP using the reverse transcriptase TGIRT for high-resolution profiling of m^1^A methylomes [35]. In addition, m^6^A-seq2 using barcoded adaptors ligated to RNAs from different samples has been developed for simultaneously interrogating m^6^A dynamics across different samples [36]. This approach could be extended to detect other RNA modifications by using their specific antibodies.

Despite the versatility of antibody-based approaches, their applications have to depend on high-quality antibodies and large quantities of starting RNAs. Alternatively, antibody-independent methods, including bisulfite sequencing (BS-seq) based on chemical-induced signature to detect m^5^C RNA modifications (Fig. 1b) [7, 37], have been developed for epitranscriptome mark detection. Sodium bisulfite treatment converts an unmethylated cytosine (C) instead of m^5^C into a uracil (U), creating a signature that allows the mapping of m^5^C at single-nucleotide resolution. However, a major disadvantage of BS-seq is an unavoidable false positive detection because of incomplete C-U conversion caused by variation of bisulfite treatment, double-stranded RNA structures, or disruption from other modifications such as hm5C [38–41]. In plants, BS-seq has been applied to detect m^5^C in tRNA, rRNA and mRNA [42–44].

Recent development of enzymatic techniques offers another useful alternative for quantitatively mapping exact locations of m^6^A in an antibody-independent manner. These techniques either take advantage of m^6^A-sensitive RNA-cleaving restriction enzymes, such as MAZTER-seq [45] and m^6^A-REF-seq [46], or utilize m^6^A erasers/readers, such as m^6^A-SEAL [47] and DART-seq [48], or make use of dimethyltransferases like MjDim1 for converting m^6^A to m^6^_2_A in m^6^A-seletive allyl chemical labeling and sequencing (m^6^A-SAC-seq) [49]. In MAZTER-seq/m^6^A-REF-seq, the m^6^A-sensitive enzyme MazF cleaves RNA at ACA rather than m^6^ACA, thus inferring quantitative site-specific m^6^A profiles (Fig. 1b). However, as MazF only recognizes the ACA motif in a subset of m^6^A sites, further engineering of the current enzymes or exploring other m^6^A-sensitive enzymes is required for expanding the toolbox. Another antibody-free approach DART-seq adopts the fusion protein of the cytidine deaminase APOBEC1 and the m^6^A-binding YTH domain APOBEC1-YTH, which induces C to U deamination at sites adjacent to m^6^As [48] (Fig. 1b). Notably, these antibody-free approaches could work on limited RNAs, as little as nanograms or even picograms, promising m^6^A profiling in rare materials or single cells. Moreover, many of them are capable of estimating the m^6^A stoichiometry across different samples. As so far these approaches have been mostly tested in mammalian systems, future optimization and application of these methods in plants will be helpful to explore context-dependent dynamics of plant m^6^A methylomes.

### Nanopore direct RNA sequencing to locate epitranscriptome marks

Although the above-mentioned epitranscriptome profiling approaches have provided unprecedented insights into the distribution and regulation of RNA modifications, challenges remain for quantitative mapping of RNA modification landscape in multiple samples. Moreover, these approaches largely depend on short-read cDNA-based sequencing which requires the conversion of RNA to cDNA. In contrast, the nanopore long-read direct RNA sequencing (DRS) platform is emerging as a promising approach to quantitatively locate and compare RNA modifications at single-nucleotide resolution across different conditions (Fig. 1b) [50, 51]. In this approach, when an RNA molecule traverses a protein nanopore, its modifications cause changes in intensity levels of the electric current, thus permitting prediction of modified bases in a quantitative manner by computational methods [52, 53]. Currently, the algorithms for predicting RNA modifications are built based on either characteristic base-calling error signatures, such as EpiNano [53] and DiffErr [31], or machine-learning methods to capture differences in raw current signals, such as Tombo [54], Nanocompore [55], and xPore [56]. Nanopore DRS and its associated algorithms have been used to profile RNA modifications, such as m^6^A, m^5^C, and ψ, in multiple organisms [56–58]. In plants, differential and comparative m^6^A methylomes at high-resolution have been generated by nanopore DRS for *Arabidopsis* mutants defective in two m^6^A writers [31, 59]. Additionally, nanopore DRS and Tombo have been used to identify m^5^C peaks in *Arabidopsis* [58], with an overall pattern similar to that identified by m^5^C-seq [19]. Future development of algorithms with a focus on improving the accuracy of detecting a broad spectrum of RNA modifications will certainly strengthen epitranscriptome studies.

Besides these experimental approaches, prediction methods, such as RAM-NPPS [60], BERMP [61], and PEA [62], have also been developed to predict m^6^A in plants. Among these methods, PEA predicts m^6^A at over 70% sensitivity and specificity in *Arabidopsis*. Together, all these detection approaches bear their own intrinsic advantages and weaknesses. For instance, different m^6^A profiling techniques require greatly varied amount of starting materials ranging from 100 ng to 20 μg of mRNA with different detection resolutions and abilities to infer stoichiometric information (Fig. 1c). These characteristics should be considered together with the biological questions to be addressed when designing an epitranscriptome profiling study. In addition, most antibody-free sequencing techniques are yet to be adopted in plant epitranscriptome research despite an increasing trend of using Nanopore DRS to locate the precise RNA modification sites in various plant species. Future applications of these profiling approaches will undoubtedly contribute to our understanding of the dynamics of plant epitranscriptome.

## Advances in characterizing epitranscriptome players in plants

### m^6^A writers and recruiters

**Fig. 2:**
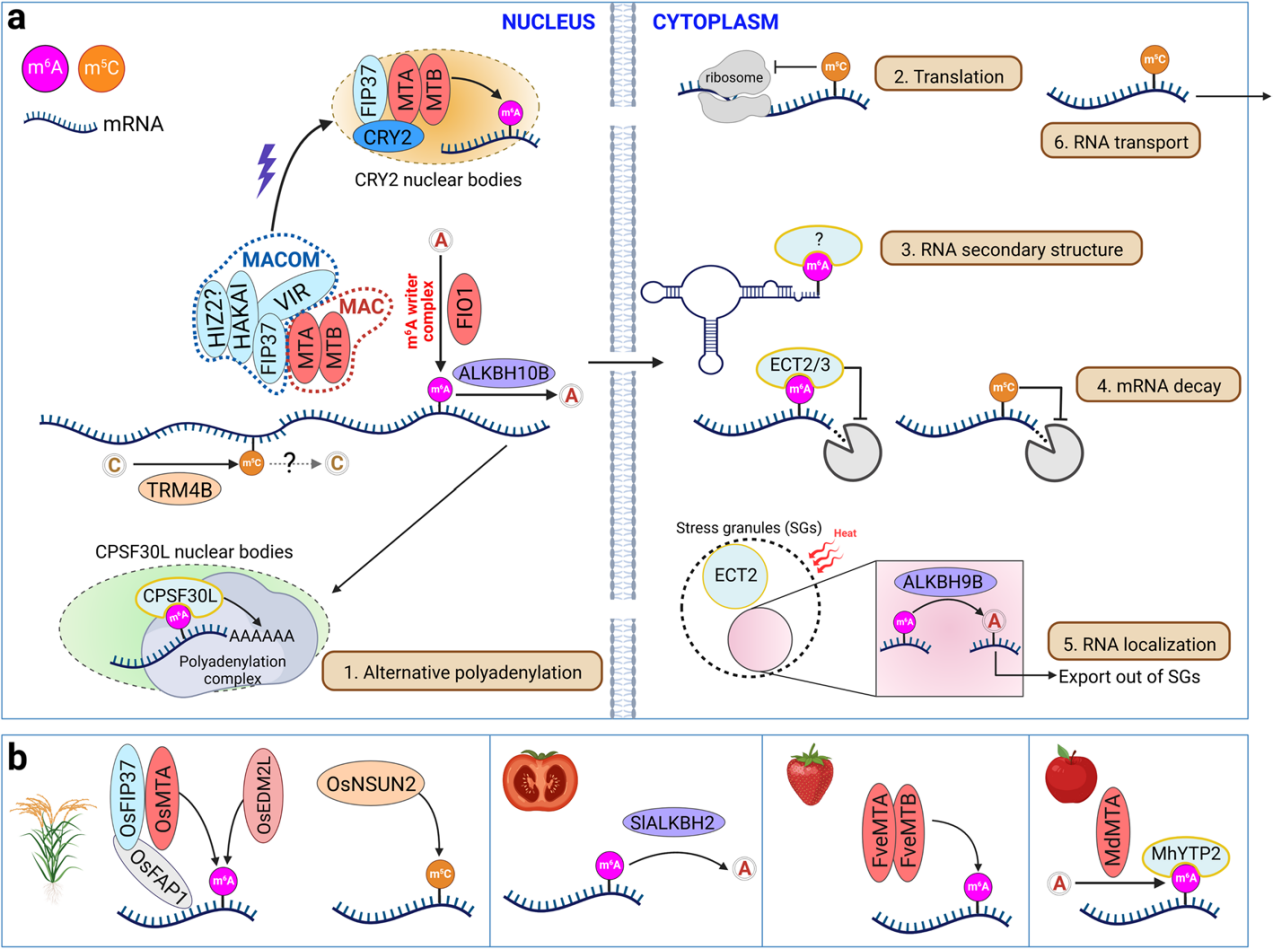
An overview of the effector proteins and molecular functions of m^6^A and m^5^C. **a** Effector proteins and molecular functions of m^6^A and m^5^C in *Arabidopsis*. m^6^A is deposited to its target transcripts mainly by a multicomponent m^6^A methyltransferase complex in the nucleus. This complex could be divided into two subcomplexes, namely the m^6^A-METTL Complex (MAC) and the m^6^A-METTL Associated Complex (MACOM). Upon blue light treatment, MTA, MTB, and FIP37 are recruited to the CRY2 nuclear bodies for m^6^A methylation of several central oscillator genes. Another known m^6^A methyltransferase FIO1 acts separately to deposit m^6^A in a subset of transcripts. m^6^A is removed by ALKBH10B in the nucleus or by ALKBH9B in stress granules (SGs) in the cytoplasm. m^6^A is recognized by CPSF30L in the nucleus or ECT2/3 in the cytoplasm. m^5^C is catalyzed by TRM4B. RNA modifications affect RNA metabolism in many aspects, including (1) alternative polyadenylation, (2) translation, (3) RNA secondary structure, (4) RNA stability, (5) RNA localization, and (6) RNA transport. **b** Some known effector proteins of m^6^A and m^5^C in crops. Created with Biorender.com

m^6^A deposition to target transcripts requires an evolutionarily conserved multicomponent m^6^A writer/methyltransferase complex. In *Arabidopsis*, this complex consists of two core methyltransferases, mRNA adenosine methylase (MTA; ortholog of METTL3) and MTB (ortholog of METTL14), and several accessory proteins including FKBP12 INTERACTING PROTEIN 37KD (FIP37; ortholog of WTAP), VIRILIZER (VIR; ortholog of VIRMA), and HAKAI (Fig. 2a and Table 1) [24, 28, 63–67]. Studies in mammals have suggested that the m^6^A methyltransferase complex could be divided into two subcomplexes, termed m^6^A-METTL Complex (MAC) and m^6^A-METTL Associated Complex (MACOM) [68]. MAC formed by MTA and MTB constitutes the catalytic core of the m^6^A methyltransferase complex, while MACOM containing the accessory subunits including FIP37, VIR, and HAKAI is required for the full activity of MAC. Several additional factors, such as RNA-binding motif protein 15 (RBM15)/RBM15B and zinc finger CCCH domain-containing protein 13 (ZC3H13), are other major components of the mammalian MACOM [69, 70]. In *Arabidopsis*, a recent study suggests that a HAKAI-interacting zinc finger protein HIZ2 might be the plant equivalent of ZC3H13 [71], but its biological function in the m^6^A writer complex needs further investigation. Additionally, although FLOWERING LOCUS PA (FPA) represents the closest ortholog of RBM15/RBM15B and co-purifies with m^6^A writers, it does not influence global m^6^A levels when it is defective or overexpressed [72], implying a limited effect of FPA on m^6^A modifications in *Arabidopsis*. Thus, whether plant m^6^A requires RBM15/RBM15B equivalents needs further exploration.

Within this m^6^A methyltransferase complex, mutual regulation among different subunits takes place especially at the post-translational level. For instance, WTAP is required for recruiting METLL3-METTL14 to nuclear speckles and mRNA targets in mammalian cells [66], and for stabilizing the METTL3-METTL14 interaction in *Drosophila* [73]. ZC3H13 facilitates nuclear localization of other m^6^A writers in mouse embryonic stem (mES) cells [69]. By contrast, the exact roles of different *Arabidopsis* m^6^A writers remain completely obscure. In particular, whether the accessory subunits, FIP37, VIR, and HAKAI, play regulatory functions in mediating m^6^A methyltransferases, MTA and MTB, awaits further examination. Nevertheless, each individual m^6^A writer, such as MTA, MTB, FIP37, and VIR, is indispensable for m^6^A deposition [28, 31, 71, 74], indicating their functional interdependence and mutual regulation for maintaining the functionality of the m^6^A methyltransferase complex in *Arabidopsis*.

This *Arabidopsis* m^6^A methyltransferase complex, like its mammalian counterpart, installs m^6^A on mRNAs preferentially near stop codons and in 3′UTRs in a major sequence context of RRACH (R = A/G; H = A/C/U) [13, 28, 31], accounting for the majority of total m^6^A levels in *Arabidopsis*. In contrast, another known methyltransferase FIONA1 (FIO1; ortholog to METTL16) acts separately to deposit m^6^A modifications on a subset of transcripts, contributing modestly to overall m^6^A levels (Fig. 2a and Table 1) [59, 75]. Unlike METLL16 association with a UACm^6^AGAGAA sequence embedded in a stem-loop structure [76, 77], FIO1-mediated m^6^A methylation is enriched in a YHAGA (Y = C/U) motif in coding sequences peaked near stop codons [59] or motifs resembling RRACH in 3′UTRs [75]. Despite these differences, both METTL16 and FIO1 deposit m^6^A to the noncoding U6 spliceosomal small nuclear RNA [59, 75, 77].

**Table 1:**
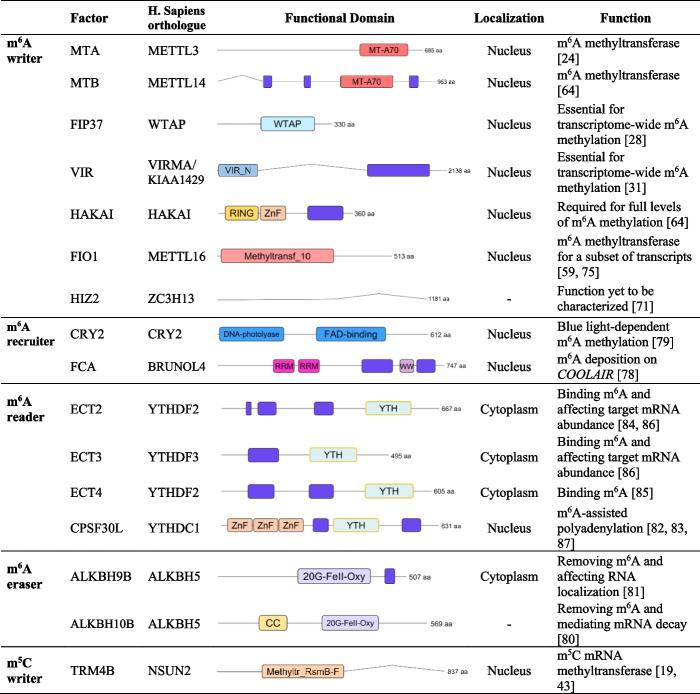
Functional domain, subcellular localization, and function of m^6^A and m^5^C effectors in *Arabidopsis* [19, 24, 28, 31, 43, 59, 64, 71, 75, 78–87]

In the “Functional domain” column, blue represents low complexity region as predicated by “Prion-like Amino Acid Composition” (PLACC) [88]; *CC*, coiled-coil domain; *ZnF*, zinc finger domain; *RRM*; RNA recognition motif

These two distinct m^6^A-depositing machineries seem to have both common and distinct targets [59, 75], raising intriguing questions regarding how m^6^A writers select their targets in response to developmental and environmental signals. Recent advances in plants suggest that target-specificity of m^6^A could be achieved through recruiting m^6^A writers to specific transcripts by RNA binding proteins (RBPs) and other writer-associated proteins, which are tentatively named m^6^A recruiters. The RBP FCA co-purifies with MTA, MTB, and FIP37 and facilitates m^6^A deposition on the noncoding antisense transcript *COOLAIR* during *Arabidopsis* flowering [78]. Another RBP OsFIP37-assocated protein 1 (OsFAP1) recruits the m^6^A writer OsFIP37 for adding m^6^A on *OsYUCCA3* transcripts during male meiosis in rice (Fig. 2b) [89]. Other characterized m^6^A recruiters include cryptochrome 2 (CRY2), which undergoes liquid-liquid phase separation (LLPS) to form CRY2-nuclear bodies in response to blue light and interacts with MTA, MTB, and FIP37 to mediate m^6^A installation on transcripts of central circadian clock oscillator genes (Fig. 2a) [79]. These studies exemplify how various factors are engaged in m^6^A-depositing machineries to achieve transcript-specific m^6^A methylation. Specific m^6^A recruiters may act at different developmental stages or under various environmental stimuli to guide m^6^A writers to distinct sets of transcripts, generating development- or stimulus-dependent m^6^A methylomes. Moreover, chromatin or epigenetic signatures also affect m^6^A deposition in mammalian cells, represented by H3K36me3 that guides m^6^A methylation co-transcriptionally through METTL14 [90]. Likewise, m^6^A sites are correlated to H3K36me2 marks in *Arabidopsis* [91], implying a potential mechanism of m^6^A deposition mediated by epigenetic signatures in plants.

Another recent study in rice has suggested the presence of a third m^6^A methyltransferase ENHANCED DOWNY MILDEW 2-like (OsEDM2L), which contains a highly conserved *N*^6^-adenine methyltransferase-like (MTL) domain (Fig. 2b) [92]. Total m^6^A levels and transcriptome-wide m^6^A enrichment are significantly reduced in *osedm2l* mutants, suggesting that OsEDM2L is indispensable for the m^6^A methylation landscape. As OsEDM2L is specifically expressed in anthers, context-dependent m^6^A methylation could be modulated by organ-specific m^6^A methyltransferases.

### m^6^A erasers

m^6^A has been known as a reversible modification since the discovery of m^6^A erasers Fat mass and obesity-associated protein (FTO) and AlkB homolog 5 (ALKBH5) [93, 94]. Although there are no plant orthologs of FTO, multiple copies of ALKBH5 orthologs have been found in various plant species [95], including six of them in *Arabidopsis*, namely ALKBH9A/9B/9C/10A/10B/10C [96]. Among ALKBH5 plant orthologs, ALKBH9B/10B in *Arabidopsis* and SlALKBH2 in tomato have been characterized to mediate m^6^A demethylation (Fig. 2 and Table 1) [80, 97, 98]. ALKBH9B not only demethylates m^6^A-containing viral RNAs and modulates viral infection [98] but also mediates demethylation of a heat-activated retroelement *Onsen* in stress granules (SGs) [81]. So far, the mechanisms by which ALKBHs select their targets for demethylation are yet to be elucidated.

### m^6^A readers

Recognition and interpretation of m^6^A by its readers affect the fate of methylated mRNAs in various mRNA metabolism processes. To date, three groups of m^6^A readers have been found to recognize m^6^A-modified transcripts through different mechanisms [99]. YTH domain-containing proteins directly bind m^6^A through highly conserved YTH domains [100, 101], while heterogeneous nuclear ribonucleoproteins (HNRNPs) recognize m^6^A-containing RNAs through m^6^A-dependent RNA structure remodeling [102–105]. Several other RBPs, such as insulin-like growth factor 2 mRNA binding protein, are associated with m^6^A-modified transcripts via unknown mechanisms [106]. By now, characterized m^6^A readers in plants all belong to the YTH domain protein family, including EVOLUTIONARILY CONSERVED C-TERMINAL REGION 2-4 (ECT2-4) and CPSF30L in *Arabidopsis* and MhYTP2 in apple (Fig. 2 and Table 1) [82–85, 107, 108].

ECT2/3/4 recognize m^6^A via their aromatic cages and function redundantly in regulating the timing of organ initiation and leaf morphology [85, 109]. In agreement with their genetic redundancy, ECT3 shares most overlapping target sites with ECT2 and modulates mRNA abundance in the cytoplasm [86]. However, there are conflicting views on functional mechanisms of ECT2. An early study suggests that ECT2 is localized both in the nucleus and cytoplasm to affect 3′UTR length and mRNA stability, respectively [84], whereas a recent study shows that ECT2 is exclusively localized in the cytoplasm to regulate its target abundance but has little direct effect on alterative polyadenylation (APA) [86]. Another m^6^A reader CPSF30L forms phase-separated nuclear bodies to influence APA of m^6^A-containing mRNAs (Fig. 2a) [82, 83, 87]. Disruption of CPSF30L results in global poly(A) site shifts and transcriptional readthrough in recently rearranged gene pairs in *Arabidopsis* [82, 87]. Additionally, the apple m^6^A reader MhYTP2 plays dual functions in mediating transcript stability and translation efficiency via unknown mechanisms [108]. Surprisingly, overexpression of *MhYTP2* leads to a transcriptome-wide increase in m^6^A levels possibly via affecting expression levels of multiple m^6^A writers and erasers, implying possible crosstalk among m^6^A effectors to maintain appropriate cellular m^6^A levels.

Notably, plant genomes encode more YTH domain proteins than other eukaryotes. For instance, there are 13 in *Arabidopsis*, 12 in rice, and 39 in wheat [95]. These genes may exhibit diverse expression patterns under different developmental and stress conditions, thus conferring functional diversities. Further exploration of their biological roles and functional modes are critical for better interpreting m^6^A epitranscriptome in plants.

### m^5^C writers

Like m^6^A, m^5^C deposition, removal, and interpretation in animals require the respective roles of writers including NOL1/NOP2/sun (NSUN) family and DNA methyltransferase homolog DNMT2, erasers such as ten-eleven translocation proteins, and readers including Aly/REF export factor and Y-box binding protein 1 [110]. However, only m^5^C writers so far have been characterized in plants and include the NSUN orthologs, such as tRNA-specific methyltransferase 4 (TRM4B), TRM4C/NOP2A, TRM4D/NOP2B, and TRM4H in *Arabidopsis* and OsNSUN2 in rice, and the DNMT2 ortholog tRNA aspartic acid methyltransferase 1 (TRDMT1) in *Arabidopsis* [19, 42, 43, 111, 112]. Among them, the *Arabidopsis* TRM4B and rice OsNSUN2 have been shown to mediate m^5^C methylation in mRNAs (Fig. 2 and Table 1).

Despite sequence homology among plant m^5^C writer proteins, m^5^C distribution patterns and targets are less conserved in various plants. For example, as revealed by BS-seq, m^5^C is evenly distributed in coding sequences (CDSs) and highly enriched in 3′UTRs in *Arabidopsis* siliques, seedling shoots and roots [43], whereas m^5^C is mostly enriched immediately after the start codon in rice seedling shoots [42]. These observations imply functional divergence of m^5^C writers in selecting their targets in different organisms. Notably, so far different m^5^C profiling approaches have revealed different m^5^C distribution patterns. For example, in contrast to those revealed by BS-seq mentioned above [43], a m^5^C-RIP-seq study uncovers strong enrichment of m^5^C in CDSs with a small peak just after the start codon and a high peak before the stop codon in *Arabidopsis* seedlings [19], while another m^5^C-RIP-seq study with a different m^5^C antibody shows enrichment of m^5^C in CDSs with a high peak after the start codon and a less pronounced peak before the stop codon [113]. Thus, it is necessary to use other approaches like miCLIP-seq [114] and Aza-IP-seq [115] to cross-check bona fide m^5^C sites in plants. In addition, as functional redundancies among members of NSUN families (e.g. eight in *Arabidopsis*) [44] confound the characterization of m^5^C writers in plants, further elucidation of their biological functions will partly rely on generation and characterization of high-order mutants.

## Advances in landscape and regulation of plant m^6^A dynamics

### Characteristics of m^6^A landscape across plant species

**Fig 3:**
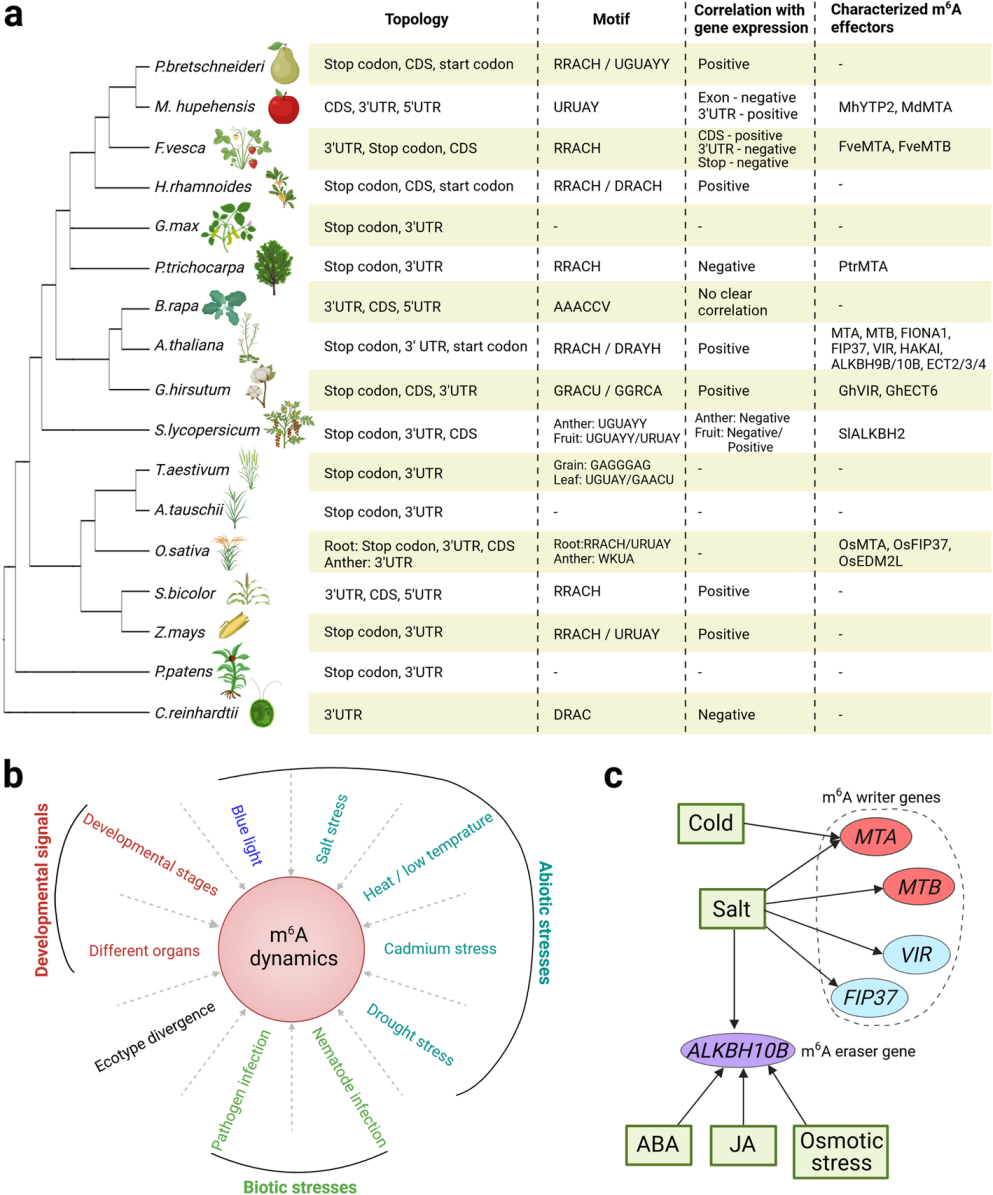
Features of m^6^A RNA methylation in plants. **a** Summary of key features of m^6^A methylation in plants. Plant species with known m^6^A profiles on a global scale are shown in a phylogenetic tree (left). The key m^6^A features (right) are summarized based on the studies in *Pyrus bretschneideri* (pear) [116], *Malus hupehensis* (apple) [108], *Fragaria vesca* (strawberry) [117], *Hippophae rhamnoides* (sea-buckthorn) [118], *Glycine max* (soybean) [27], *Populus trichocarpa* (populus) [119–121], *Brassica rapa* (pak-choi) [122], *Arabidopsis thaliana* (*Arabidopsis*) [28, 29], *Gossypium hirsutum* (cotton) [133, 136], *Solanum lycopersicum* (tomato) [97, 123, 124], *Triticum aestivum* (common wheat) [125, 126], *Aegilops tauschii* (rough-spike hard grass) [27], *Oryza sativa* (rice) [92, 127], *Sorghum bicolor* (sorghum) [128], *Zea mays* (maize) [134], *Physcomitrella patens* (earthmoss) [27], and *Chlamydomonas reinhardtii* (green algae) [130]. Among these features, the “correlation with gene expression” is shown at the transcriptome-wide basis. R = A/G; W = A/U; K = G/U; Y = C/U; D = A/G/U; H = A/C/U; V = A/G/C. **b** m^6^A modifications are influenced by various endogenous and environmental signals as shown in the following studies: developmental stages (strawberry [117], wheat [125]); different organs (*Arabidopsis* [137]); ecotype divergence (*Arabidopsis* [138]); blue light (*Arabidopsis* [79]); salt stress (*Arabidopsis* [139, 140], sweet sorghum [128], rice [141], sugar beet [142], cotton [133]); heat/low temperature (pak-choi [122], *Arabidopsis* [143], tomato [124]); cadmium stress (rice [127], barley [132]); drought stress (sea-buckthorn [118], populus [119], apple [135, 144]); pathogen infection (apple [108], rice [145], wheat [126], watermelon [131], pear [116]); and nematode infection (soybean [146]). **c** Expression of m^6^A writer and eraser genes are modulated by multiple external stimuli in *Arabidopsis*. Arrows indicate positive regulation. Created with Biorender.com

Transcriptome-wide m^6^A targets and distribution have been extensively profiled with m^6^A-seq or nanopore DRS in multiple plant species, including pear, apple, strawberry, soybean, pak-choi, *Arabidopsis*, tomato, rice, maize, etc. [27–29, 92, 97, 108, 116–134]. m^6^A methylation ratios range from 29% in common wheat to 51% in earthmoss across 13 plant species [27]. The m^6^A distribution preference around stop codons and in 3′UTRs is highly conserved across most plant species from green algae to higher land plants (Fig. 3a), indicating that m^6^A is an evolutionarily conserved RNA modification. Despite their low abundance, m^6^A marks in CDSs have been consistently observed in several plant species, such as pear, strawberry, sea-buckthorn, pak-choi, and rice [92, 116–118, 122, 127]. In the rare case, m^6^A is highest enriched in CDSs in apple leaf [108]. These observations indicate that m^6^A in CDS might be a previously neglected but important feature with functional significance. For instance, there is an increase in m^6^A peaks in CDSs during strawberry fruit ripening [117], implying that m^6^A in CDS might be associated with changing developmental contexts. In addition, m^6^A enrichment is also observable around the start codon in *Arabidopsis*, pear, and sea-buckthorn [28, 116, 118] and within 5’UTRs in apple, pak-choi and sweet sorghum [122, 128, 135].

m^6^A mainly falls into two sequence motifs in various plant species, including the conserved RRACH motif and the plant-specific URUAY (Y = C/U) motif, whereas a distinct AAACCV (V = A/G/C) motif has only been reported in pak-choi (Fig. 3a) [122]. Interestingly, m^6^A could occur in divergent sequence contexts at different developmental stages of the same plant species. For instance, in common wheat, the GAGGGAG and UGUAY motifs are found in m^6^A peaks in grains and leaves, respectively [125, 126].

### Dynamic regulation of m^6^A distribution

m^6^A methylomes are dynamically changed at different developmental stages and in response to environmental stimuli in diverse plant species (Fig. 3b). Among different *Arabidopsis* tissues including roots, rosette leaves, and flowers, the fraction of transcripts displaying differential m^6^A modifications is significantly larger than that showing different transcript levels [137], indicating that m^6^A may contribute to organ differentiation.

m^6^A modifications are also dynamically affected by various stresses. Abiotic stresses, such as salt, drought, and heat, or biotic stresses, such as virus and fungal diseases, do not significantly influence the overall m^6^A distribution pattern in the 3′UTR and around stop codons but greatly induce dynamic m^6^A redistribution on selected transcripts [79, 108, 117–119, 122, 124–128, 131–133, 135, 137–147]. Salt stress significantly increases m^6^A methylation in *Arabidopsis* seedlings [139] and rice shoots but not in rice roots [141]. It induces dynamic deposition of m^6^A to salt-stress-related transcripts to protect them from degradation in *Arabidopsis* [140] and also increases m^6^A methylation on some salt-resistant-related transcripts to enhance their RNA stability in sweet sorghum [128]. Drought stress causes changes in m^6^A levels of drought-responsive genes, thereby affecting their expression levels in apple [135]. Cadmium (Cd) induces a transcriptome-wide m^6^A hypermethylation in barley roots [132] and alters methylation levels of a large number of transcripts in rice [127]. Together, these observations imply a prominent role of stress-induced m^6^A redistribution in stress adaptation.

Although many studies have revealed dynamic m^6^A methylations in organ-, age-, and stress-dependent manners in plants, the underlying mechanisms so far remain elusive. Such dynamic m^6^A regulation in various contexts could be partially achieved through titration of levels of m^6^A writers and erasers, resulting in global m^6^A redistribution. In *Arabidopsis*, salt stress increases m^6^A methylation likely through upregulating the expression of the m^6^A writer genes, *MTA*, *MTB*, *VIR*, and *FIP37* (Fig. 3c) [139]. Interestingly, *ALKBH10B* expression is also upregulated under salt stress [148], indicating that salt stress-induced m^6^A dynamics is cooperatively sculpted by increased levels of m^6^A writers and erasers. Drought stress reduces m^6^A methylation in sea-buckthorn, which is associated with a significantly increased expression of m^6^A demethylase genes *HrALKBH10B/10C/10D* [118]. Besides transcriptional regulation, post-transcriptional modification of m^6^A effectors or deployment of m^6^A recruiters under various conditions may also contribute to m^6^A dynamics. For instance, upon blue light treatment, MTA, MTB, and FIP37 are recruited to the CRY2 nuclear bodies for selective m^6^A methylation on central oscillator transcripts [79]. Further exploring the mechanisms underlying dynamic m^6^A alterations on selective transcripts under various conditions will advance our mechanistic understanding of context-dependent regulation of m^6^A dynamics.

## Advances in plant epitranscriptome in gene regulation

**Fig. 4:**
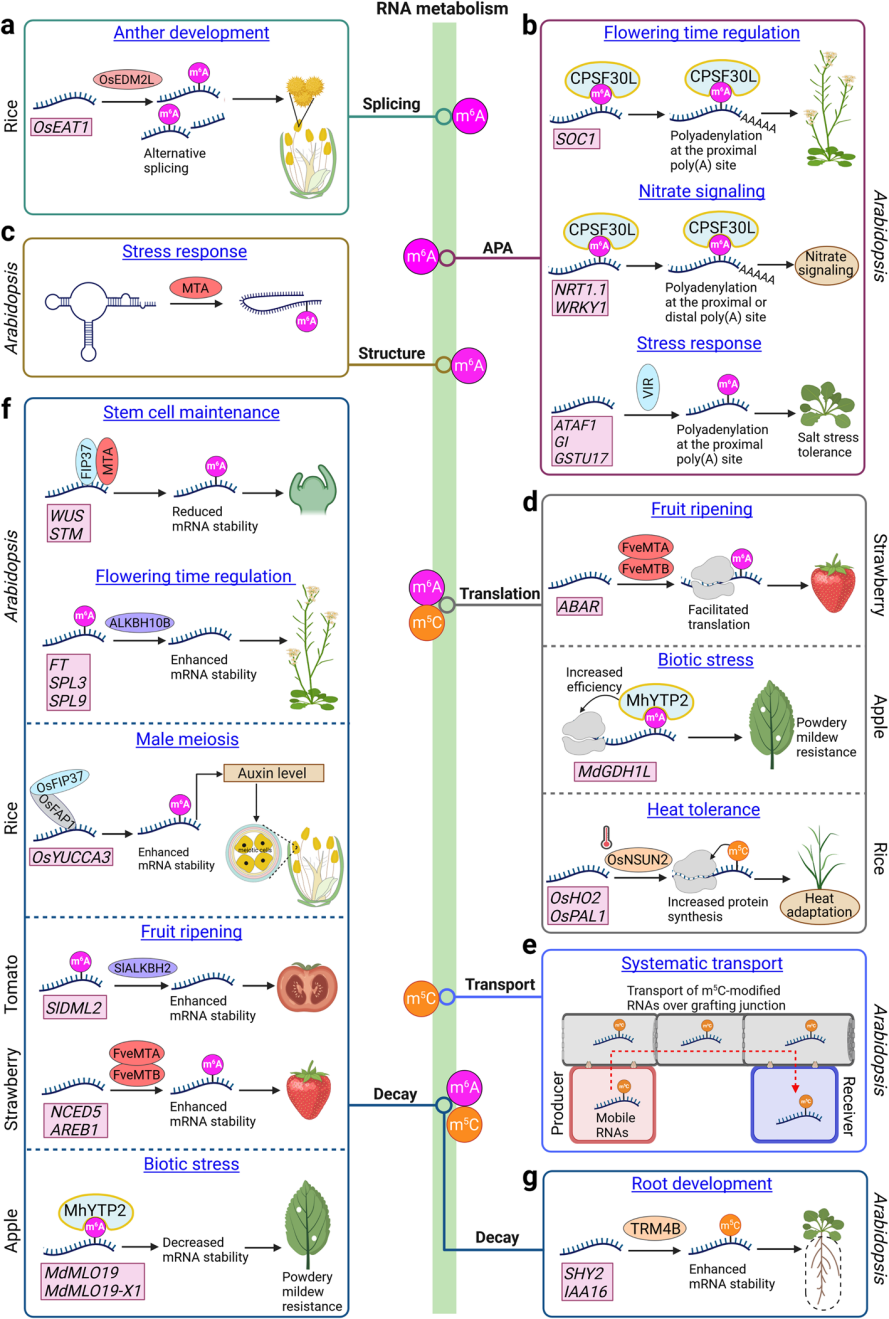
Epitranscriptome-mediated RNA metabolism and its effects on plant development, cellular processes, and stress responses. **a** OsEDM2L mediates m^6^A modification of *OsEAT1*, resulting in proper alternative splicing of *OsEAT1* in rice anther development. **b** m^6^A modification affects alternative polyadenylation (APA) in *Arabidopsis*. Binding of CPSF30L to m^6^A-modified *SOC1* mRNA regulates its APA and results in relatively stable *SOC1* transcripts with a shorter 3′UTR to promote flowering. CPSF30L also mediates nitrate signaling through regulating the APA of several m^6^A-modified transcripts, including *NRT1.1* and *WRKY1*, in the nitrate signaling pathway. VIR mediates m^6^A modification and APA of several stress-related transcripts in salt stress response. **c** m^6^A deposition on salt-stress-responsive transcripts by MTA is associated with a decrease in RNA secondary structures, causing increased RNA stability. **d** m^6^A modification affects protein translation in several crops. In strawberry fruit ripening, FveMTA- and FveMTB-mediated m^6^A modification of *ABAR* facilitates its translation. In apple, binding of MhYTP2 to m^6^A-modified *MdGDH1L* promotes its translation to confer powdery mildew resistance. In rice, OsNSUN2-dependent m^5^C modification increased protein synthesis to enhance rice adaptation to heat stress. **e** m^5^C RNA modification regulates RNA transport over the grafting junction in *Arabidopsis*. **f** m^6^A modification affects RNA stability in various plants. In *Arabidopsis*, m^6^A modification of *WUS* and *STM* mediated by FIP37 and MTA reduces their mRNA stability to maintain normal stem cell activity. The m^6^A eraser ALKBH10B demethylates *FT*, *SPL3*, and *SPL9*, thus enhancing their mRNA stability to promote flowering in *Arabidopsis*. In rice, OsFIP37 interacts with OsFAP1 to deposit m^6^A modification on *OsYUCCA3* transcripts to promote auxin biosynthesis required for male meiosis. In tomato fruits, SlALKBH2 demethylates and enhances the stability of *SlDML2* to accelerate fruit ripening. In strawberry, FveMTA and FveMTB deposit m^6^A modification on *NCED5* and *AREB1* transcripts, thus enhancing their RNA stability to promote fruit ripening. In apple, MhYTP2 binding to m^6^A-modified transcripts of *MdMLO19* and *MdMLO19-X1* destabilizes their transcripts to promote resistance to powdery mildew. **g** TRM4B-dependent m^5^C modification enhances RNA stability of its target transcripts in *Arabidopsis* root development. Created with Biorender.com

RNA modifications determine plant mRNA fate through influencing various aspects of mRNA metabolism, including alternative splicing (AS), APA, folding, translation, localization, transport, and decay (Figs. 2a and 4) [13, 15, 16]. These effects on mRNA metabolism ultimately impact a wide range of physiological processes in plant development and stress responses, as demonstrated by characterization of a collection of the mutants defective in RNA modification effectors.

### m^6^A function in the nucleus

m^6^A affects the pre-mRNA maturation processes, including AS and APA, to different extents in plants. Although decreased m^6^A methylation in *FIP37*-, *VIR*-, or *FIO1*-defective *Arabidopsis* mutants [28, 31, 59, 75] or *OsFIP37*-defective rice [149] has a mild effect on transcriptome-wide AS, OsEDM2L-mediated m^6^A methylation on *ETERNAL TAPETUM 1* (*OsEAT1*) affects AS of *OsEAT1*, thereby modulating rice anther development (Fig. 4a) [92]. These observations indicate that instead of having a general effect on AS, m^6^A may regulate AS in specific transcripts or tissues. In contrast, m^6^A influences APA on a transcriptome-wide scale. Loss of VIR-mediated m^6^A results in defective 3′end formation of RNAs, which is mainly a shift to usage of proximal poly(A) sites [31]. CPSF30L regulates global poly(A) site choice through recognizing m^6^A-modified far-upstream sequence but does not show clear preference to proximal or distal poly(A) sites [82]. This m^6^A-assisted polyadenylation by CPSF30L also restricts transcription readthrough and chimeric mRNA formation at rearranged genomic loci, thus protecting transcriptome integrity [87]. A correlation of m^6^A sites with APA has also been observed in maize and populus [119, 120], implying a general role of m^6^A in regulating APA in plants. Moreover, APA at specific transcripts has been linked with plant physiological processes (Fig. 4b). For instance, CPSF30L modulates APA of several transcripts, including *SUPPRESSOR OF OVEREXPRESSION OF CONSTANS 1* (*SOC1*) and *NRT1.1* to influence floral transition and nitrate signaling [82, 83]. VIR-mediated m^6^A methylation affects 3′UTR lengthening via APA of several salt stress regulators including *ATAF1*, *GI*, and *GSTU17* in stress adaptation [139].

Besides pre-mRNA maturation, accumulating evidence suggests that m^6^A exerts important roles in regulating chromatin accessibility and transcription in mammalian cells [150, 151], whereas the similar evidence has just begun to emerge in plants. m^6^A affects dynamics of FCA nuclear condensates, further influencing the DNA:RNA hybrid (R-loop) formation at the *COOLAIR* locus in *Arabidopsis* [78], which indicates a possible role of m^6^A in chromatin regulation. Moreover, overexpression of the human *FTO* in rice increases chromatin accessibility, resulting in a more open chromatin state [152]. Whether m^6^A mediated by endogenous m^6^A effectors in plants modulates chromatin states needs further exploration.

### m^6^A function in the cytoplasm

Compared with its nuclear roles in pre-mRNAs, cytoplasmic roles of m^6^A in affecting mature mRNA metabolism, including folding, translation, localization, and decay, are better understood (Figs. 2a and 4). m^6^A deposition on transcripts in salt-dependent manner is negatively correlated with RNA folding and the resulting secondary structure, which further affects RNA stability (Fig. 4c) [153]. The m^6^A-mediated change in RNA secondary structure has also been observed in primary microRNAs (pri-miRNAs), in which MTA-mediated m^6^A deposition induces their secondary structures [154]. These observations imply that m^6^A has both stimulatory and inhibitory effects on RNA folding, which may be target- or context-dependent. Structural change in RNAs induced by m^6^A may affect their interactions with RNA-binding proteins [104], thus influencing other RNA metabolism steps.

Recent studies have also shown m^6^A effect on mRNA translation in plants. Combination of m^6^A-seq and polysome profiling analyses in maize has revealed complex correlations between m^6^A and translation efficiency, in which m^6^A is negatively correlated with translation status on the global scale, whereas m^6^A close to the start codon tends to facilitate translation [155]. Similarly, a m^6^A site in the 5’UTR of *Md4CL3* transcripts in apple has been shown to promote translation [135], which is similar to the observation in HeLa cells [156]. The promotive effect of m^6^A on translation has also been observed in apple transcripts containing m^6^A in the 3′UTR and the transcripts in strawberry fruit [108, 117]. Moreover, m^6^A-mediated translation seems to have important functions in different physiological processes (Fig. 4d). FveMTA/FveMTB-mediated m^6^A methylation on the putative ABA receptor (*ABAR*) facilitates translation of *ABAR*, thereby regulating strawberry fruit ripening [117], while the apple m^6^A reader MhYTP2 enhances translation of *MdGDH1L*, conferring resistance to powdery mildew [108]. Despite these observations, the mechanisms underlying m^6^A role in translation remain unknown in plants.

So far, the effect on mRNA stability is the best characterized role of m^6^A in various plant species and influences multiple aspects of developmental processes and stresses responses. From a transcriptome-wide perspective, m^6^A displays both promotive and inhibitory effects on mRNA stability in plants. In *Arabidopsis*, loss of MTA-, VIR-, and FIP37-dependent m^6^A methylation leads to a global reduction of transcript abundance [28, 31, 140], indicating a stabilizing role of m^6^A in mRNA. Likewise, m^6^A readers ECT2/3 stabilize transcripts as most target transcripts of ECT2/3 exhibit reduced abundance upon loss of *ECT2/3/4* [86]. These effects on increasing RNA stability likely result from the inhibition of ribonucleolytic cleavage by m^6^A [140]. Effects of m^6^A on gene expression have also been examined in various crops via combining m^6^A-seq and RNA-seq (Fig. 3a). Positive correlation between m^6^A and transcript abundance has been similarly observed in pear [116], sea buckthorn [118], cotton [133], salt-resistant related transcripts in sweet sorghum [128], and maize genes bearing 2,4-D-induced m^6^A peaks [129], whereas negative correlation has been shown in populus [120] and green alga [130]. Interestingly, m^6^A and transcript abundance are generally negatively associated in anthers and during fruit ripening [97, 124] but positively correlated during fruit expansion in tomato [123]. Moreover, m^6^A near the stop codon and within the 3′UTR is negatively related to transcript abundance, whereas m^6^A in the CDS tends to stabilize mRNA in strawberry fruit [117]. These complex relationships between m^6^A and transcript abundance reflect distinct cellular fates of m^6^A-modified mRNAs in different contexts, implying that mRNA decay mediated by m^6^A is likely affected by species, developmental stage, cell type, and stress. However, as previous studies have been mostly focused on entire seedlings or organs [157], the heterogeneity of m^6^A dynamics and its effect on transcript abundance so far remain elusive. Thus, further analyses, including measuring the half-life of RNA at the transcriptome-wide scale, in various tissues and developmental stages of defective mutants of m^6^A writers, erasers, or readers will be necessary to disclose the complex and dynamic effects of m^6^A on transcript stability.

It has also been shown that m^6^A exerts promotive and inhibitory roles on stability of specific transcripts, which is of great importance in regulating many biological processes in plants (Fig. 4f). In *Arabidopsis*, FIP37-dependent m^6^A deposition on *WUSCHEL* (*WUS*) and *SHOOTMERISTEMLESS* (*STM*) mRNAs accelerates their mRNA decay, which is crucial for maintaining the normal function of shoot apical meristem [28]. The m^6^A demethylase ALKBH10B demethylates m^6^A-containing transcripts of *FLOWERING LOCUS T* (*FT*) and *SQUAMOUSA PROMOTER BINDING PROTEIN-LIKE 3/9* (*SPL3/9*), thereby increasing their mRNA stability to promote flowering [80]. Likewise, the tomato SlALKBH2 demethylates the m^6^A-modified *SlDML2* transcript and increases its stability, contributing to fruit ripening [97]. Overexpression of an apple m^6^A reader MhYTP2 promotes the decay of *MdMLO19* and *MdMLO19-X1*, thus promoting apple resistance to powdery mildew [108]. These studies exemplify how m^6^A destabilizes specific transcripts to regulate various developmental processes and stress responses. By contrast, m^6^A also stabilizes specific transcripts in multiple physiological processes. In rice, m^6^A methylation on *OsYUCCA3* mediated by the OsFIP37-OsFAP1 complex stabilizes *OsYUCCA3* transcripts, thereby promoting local auxin biosynthesis in anthers to secure successful male meiosis and fertility [89]. In strawberry, FveMTA-mediated m^6^A modifications on *NCED5* and *AREB1* enhance their transcript stability to ensure normal fruit ripening [117].

While these pieces of evidence clearly demonstrate that m^6^A affects transcript stability, the underlying regulatory modes await further examination. Recognition of m^6^A marks by different m^6^A readers is possibly important for sorting mRNAs for stabilization or destabilization. It has been suggested that in mammalian cells, YTHDF2 (ortholog of ECT2/3/4) mediates degradation of m^6^A-containing transcripts via two mechanisms: directly recruiting the CCR4-NOT deadenylase complex for deadenylation of m^6^A-containing transcripts or interacting with endoribonucleases for endoribonucleolytic cleavage [158–160]. On the contrary, expression levels of a majority of ECT2/3-target RNAs are decreased in *ect2/3/4* triple mutants, indicating the roles of ECT2/3 in stabilizing transcripts [86]. Intriguingly, upon heat exposure, ECT2 is relocated to SGs through which ECT2 may affect transcript stability [107]. Similarly, the complexes of YTHDFs-m^6^A-marked mRNAs are partitioned into phase-separated compartments including SGs under stress conditions in mES cells [161]. Many plant ECTs contain low complexity regions or prion-like domains (PrLDs), which could drive LLPS to facilitate formation of membraneless condensates (Table 1) [82, 162], implying that interaction between ECTs and their associated m^6^A-modified RNAs in distinct cellular compartments might be important for determining the stability of these RNAs.

In addition to the above cytoplasmic roles, m^6^A may also function in regulating RNA localization. m^6^A-demethylation of the heat-activated retroelement *Onsen* mediated by SG-localized ALKBH9B releases *Onsen* from SG, thus altering its localization (Fig. 2a) [81]. Taken together, current studies have suggested multifaceted nuclear and cytoplasmic roles of m^6^A in post-transcriptional gene regulation and also raised intriguing questions about the fundamental nature of the relevant mechanisms.

### m^5^C function

As another abundant internal mRNA modification, m^5^C has also been studied, to a lesser extent, in recent years, which uncovered its important roles in mediating RNA translation, transport, and stability. m^5^C is associated with mRNAs with low translational activity in *Arabidopsis* [19], whereas m^5^C mediated by OsNSUN2 facilitates protein synthesis in rice [42], indicating the functional divergence of m^5^C in different plant species. In particular, OsNSUN2-mediated m^5^C on several targets, including *OsHO2* and *OsPAL1*, promotes their protein synthesis, thus enhancing rice adaptation to heat stress (Fig. 4d) [42]. Interestingly, heat stress leads to increased m^5^C methylation on mRNAs involved in photosynthesis and detoxification systems [42], implying that m^5^C is dynamically modulated during stress conditions.

In *Arabidopsis*, the m^5^C methyltransferase TRM4B promotes root growth by enhancing mRNA stability of its methylation targets, such as *SHORT HYPOCOTYL 2* (*SHY2*) and *INDOLEACETIC ACID-INDUCED PROTEIN 16* (*IAA16*) (Fig. 4g) [19, 43]. In addition, m^5^C is highly enriched in mobile mRNAs [58, 113]. TRM4B- and TRDMT1-mediated m^5^C modifications on mobile mRNAs are essential for systematic mRNA transport from producer to receiver cells over graft junctions (Fig. 4e) [113].

## Outlook

The last decade has witnessed rapid advances in plant epitranscriptome with respect to m^6^A dynamics under stresses in different plant species and mechanistic understanding of m^6^A and m^5^C modifications in the model plant *Arabidopsis* and rice. Current evidence strongly suggests that epitranscriptomic marks constitute an essential layer of post-transcriptional gene regulation that determines mRNA fate and ultimately influences plant development and adaptation to various environmental stresses. However, our understanding of plant epitranscriptome is still in its infancy. Many open questions regarding the target selectivity and functional modes of epitranscriptome marks remain to be explored. For example, how do writers and erasers select their targets in different physiological contexts? How are mRNA modifications dynamically regulated in response to environmental stimuli? How do reader proteins recognize their targets and exert their roles in subsequent RNA metabolic processes? Furthermore, since RNA modifications are highly dependent on cellular contexts, the relevant regulatory pathways may differ in different tissues and organs, at distinct developmental stage, or under different stresses. Thus, it is necessary to analyze RNA modification dynamics from tissue levels to cellular levels at single-nucleotide resolution via newly developed profiling techniques. This will advance and expand our knowledge in the plant epitranscriptome.

Multifaced roles of epitranscriptome in developmental processes and stress adaptations in diverse plant species underpin that editing epitranscriptome is a promising strategy for crop improvement. Indeed, introducing the human m^6^A demethylase in rice and potato not only increases yield but also enhances drought tolerance [152], demonstrating empirically the great potential of epitranscriptome editing in boosting agricultural production. In particular, advanced DNA/RNA editing techniques in recent years facilitate the development of multiple strategies for epitranscriptome editing of crops [63, 163]. First, modulation of expression or activity of RNA modification-related proteins (RMPs) could be achieved through CRISPR/Cas9-mediated gene editing, thus affecting traits mediated by RNA modifications. Second, RNA modification sites could be directly mutated on specific targets through precise base editors, such as the adenine base editor composed of the catalytically inactive CRISPR/Cas9 protein and an engineered adenosine deaminase causing A to G substitution [164, 165]. Third, RNA modifications could be specifically created or removed on specific target sites through the catalytically inactivated Cas13 (dCas13)-RMPs. For examples, in mammalian cells, dCas13 fused with a truncated methyltransferase domain of METTL3 guides site-specific m^6^A methylation in target transcripts [166], while dCas13 fused to m^6^A demethylases, such as ALKBH5, results in targeted RNA demethylation [167]. Intriguingly, targeted RNA demethylation or methylation can also be achieved in spatiotemporal manners using dCas13 fused with the light-sensitive protein CBIN and its adaptor CRY2 linked to FTO or essential domains of METTL3/14, respectively [168].

It is noteworthy that the application of epitranscriptome editing in crop biotechnology should at least address the following bottlenecks. First, as m^6^A sites at single-base resolution are largely unknown in crops, it is necessary to apply techniques, such as miCLIP, MAZTER-seq, m^6^A-SAC-seq, and Nanopore DRS, to precisely interrogate m^6^A at single-nucleotide resolution in crops. Second, biological effects of removing/adding epitranscriptome mark at a specific transcript are mostly unknown in plants. Third, although writers, erasers, and readers of RNA modifications have been identified in diverse crops [95], their endogenous roles and functional mechanisms remain largely elusive. Fourth, DNA/RNA-editing systems and their target specificities and editing efficiencies have yet to be established and examined in most crops. Thus, precise identification of RNA modification sites through advanced profiling approaches, mechanistic understanding of epitranscriptome marks, and development of efficient plant DNA/RNA editors should constitute integral parts of epitranscriptome editing to maximize its potential for crop improvement.

## Supplementary Information

Additional file 1. Review history.

## Data Availability

Not applicable.
