# Supplementary material for: Recent advances in the plant epitranscriptome
Source: Genome Biol. 2023 Mar 7;24:43. doi: 10.1186/s13059-023-02872-6 (PMC9990323; doi:10.1186/s13059-023-02872-6)
Supplement: Supplementary file 1 — Additional file 1. Review history. [file 13059_2023_2872_MOESM1_ESM.docx]

Review History

**First round of review**

**Reviewer 1**

**Comments to author:**

This is a very nice and timely review focused on the epitranscriptome marks m6A and m5C and their identification and effects in plant transcriptomes. The authors do a very nice job of covering all the recent findings for these marks in plant systems. My two major suggestions for improving this review manuscript are the following. First, the authors should give a bit more interpretation of the findings and approaches they cover. What findings are debatable versus those that have been consistent in the field. Furthermore, what approaches are best to use to address different research questions. A bit more author-based discussion and analysis need to be part of a high-level review article, and this is mostly missing in this document. Lastly, it would be great if the authors could expand a bit more on thoughts and future directions for utilizing these (and other covalent RNA modification systems) for crop improvement purposes. This is highlighted as a major theme of the review in the Abstract and I felt this aspect was interesting but a bit lost at the end of the manuscript with very little depth. More addition in this area would improve the impact of this review in my opinion.

**Reviewer 2**

**Comments to author:**

Epigenetic modifications are common and have been shown to regulate plant growth, fruit development, and responses to fungal and viral stresses. The most common mRNA modifications are N1-methyladenine (m1A), 5-methylcytosine (m5C), and N6-methyladenine (m6A). The authors begin with a good review of the current status of the field in characterizing plant epitranscriptome and its regulatory mechanisms, and some of the recent advances for epitranscriptome editing in crop genetic improvement. This review may advance the knowledge in the plant epitranscriptome. Thus, I fully recommended to publish this manuscript in Genome Biology after minor revisions.

Minor concerns,

Figure 1 should be embellished.

**Authors Response**

**Point-by-point responses to the reviewers’ comments:**

Reviewer #1:

This is a very nice and timely review focused on the epitranscriptome marks m6A and m5C and their identification and effects in plant transcriptomes. The authors do a very nice job of covering all the recent findings for these marks in plant systems. My two major suggestions for improving this review manuscript are the following. First, the authors should give a bit more interpretation of the findings and approaches they cover. What findings are debatable versus those that have been consistent in the field. Furthermore, what approaches are best to use to address different research questions. A bit more author-based discussion and analysis need to be part of a high-level review article, and this is mostly missing in this document. Lastly, it would be great if the authors could expand a bit more on thoughts and future directions for utilizing these (and other covalent RNA modification systems) for crop improvement purposes. This is highlighted as a major theme of the review in the Abstract and I felt this aspect was interesting but a bit lost at the end of the manuscript with very little depth. More addition in this area would improve the impact of this review in my opinion.

*Reply: We thank the reviewer for the encouraging comments and suggestions for improving the manuscript. We have revised the manuscript according to these suggestions. First, we have provided more interpretation and our own thought, when necessary, on the findings and approaches reviewed in this manuscript. These include some discussions on the debatable findings in the field. We have also generated a new Figure 1c to compare the key features of different profiling approaches that are helpful for designing various epitranscriptome profiling studies. Second, we have expanded our discussion on the potentials and challenges pertaining to utilization of epitranscriptome editing in crop improvement.*

Reviewer #2

Epigenetic modifications are common and have been shown to regulate plant growth, fruit development, and responses to fungal and viral stresses. The most common mRNA modifications are N1-methyladenine (m1A), 5-methylcytosine (m5C), and N6-methyladenine (m6A). The authors begin with a good review of the current status of the field in characterizing plant epitranscriptome and its regulatory mechanisms, and some of the recent advances for epitranscriptome editing in crop genetic improvement. This review may advance the knowledge in the plant epitranscriptome. Thus, I fully recommended to publish this manuscript in Genome Biology after minor revisions.

Minor concerns,

Figure 1 should be embellished.

*Reply: We thank the reviewer for this suggestion and have embellished Figure 1 (e.g. changing panel background and font color) in the revised manuscript.*

**Second round of review**

**Reviewer 1**

This manuscript is improved and addresses my comments. There are some minor grammatical errors throughout the manuscript that should be corrected before publication but this can be done with minor revisions. No further review is necessary by me.
